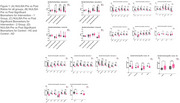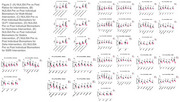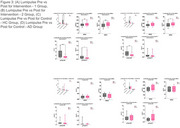# A Blood Biomarker‐guided Precision Medicine Approach for Individualized Neurodegenerative Disease Risk Reduction and Treatment: The Future of Preventive Neurology?

**DOI:** 10.1002/alz70861_108643

**Published:** 2025-12-23

**Authors:** Christopher M Janney, John G. Westine, Kellyann Niotis, Shannon Helfman, Nicholas Clute‐Reinig, Sara Murray, Hollie Hristov, Jannatul Dishary, Danny Angerbauer, Corey Saperia, Alon Seifan, Juan Melendez, Jessica P. Lakis, Licet Valois, Chelsea Brubeck, Skylar Olson, Larissa Silva, Praveen Parthasarathy, Helena Colvee, Philip Sisser, Beth Lewis, Maia Mosse, Diana Saville, Audree Rumberger, Michael McCullough, Richard S. Isaacson

**Affiliations:** ^1^ The Institute for Neurodegenerative Diseases (IND) Florida, Boca Raton, FL USA; ^2^ Institute for Neurodegenerative Diseases (IND), Boca Raton, FL USA; ^3^ The Institute for Neurodegenerative Diseases (IND), Boca Raton, FL USA; ^4^ The Atria Institute, New York, NY USA; ^5^ Jersey Memory Assessment Service, St Helier, UK Jersey; ^6^ Renaissance School of Medicine at Stony Brook University, Stony Brook, NY USA; ^7^ University of Miami School of Nursing and Health Studies, Miami, FL USA; ^8^ FAU Charles E. Schmidt College of Medicine, Boca Raton, FL USA; ^9^ Stanford University School of Medicine, Stanford, CA USA; ^10^ BrainMind, Cambridge, MA USA; ^11^ BrainMind, Watertown, MA USA; ^12^ University of California, San Francisco School of Medicine, San Francisco, CA USA; ^13^ Institute for Neurodegenerative Diseases (IND) Florida, Boca Raton, FL USA

## Abstract

**Background:**

This study investigates plasma proteins as potential markers for early detection and intervention of Alzheimer's Disease (AD) and other Neurodegenerative Diseases (NDDs). Participants with a family history of NDDs and minimal neurological symptoms, along with healthy controls, were recruited from five sites in the US and Canada. As of April 23, 2025, 198 participants were recruited, with 81 having longitudinal assessments analyzed.

**Method:**

Participants receiving preventive neurology or medicine care were divided into two groups: "Intervention 1" for those adhering to over 60% of risk reduction interventions, and "Intervention 2" for those adhering to less than 60%. These were compared to healthy controls and AD controls. NDD risk reduction included lifestyle changes, lipid‐lowering agents, GLP1s, HRT, Statins, Zetia, and SSRIs.

**Results:**

NULISA testing revealed significant changes in three ratios (Aβ42/40, pTau217/Aβ42, pTau181/Aβ42, Oligo‐SNCA/SNCA) for Intervention 1 and two ratios for Intervention 2. Additionally, 34 individual biomarkers changed significantly in Intervention 1 and 26 in Intervention 2. Multi‐modal interventions showed the highest number of significant changes.

Lumipulse testing showed significant differences in the pTau181/AB42 ratio, Aβ40, Aβ42, pTau181, and pTau217 in Intervention 1, and changes in the pTau181/AB42 ratio, Aβ42, and pTau181 in Intervention 2. Controls showed no significant changes.

**Conclusion:**

The study concluded that higher compliance to interventions led to more significant changes in protein markers. Multi‐modal interventions were most effective. Novel alpha‐synuclein markers also changed, potentially aiding in evaluating interventions for Lewy Body Dementia and Parkinson's disease. These markers may serve as future outcome measures for preventive neurology care.